# NEIGHBORHOOD ENVIRONMENT AND COGNITIVE FUNCTION AMONG OLDER ADULTS IN BRAZIL

**DOI:** 10.1093/geroni/igae098.3122

**Published:** 2024-12-31

**Authors:** Lourdes Romañach Álvarez, Mateo Farina, Elizabeth Muñoz

**Affiliations:** The University of Texas Austin, Austin, Texas, United States; University of Texas at Austin, Austin, Texas, United States; The University of Texas Austin, Austin, Texas, United States

## Abstract

Neighborhood characteristics, such as social cohesion and community resources, are linked to better cognitive functioning in older adulthood. However, little is known about the significance of neighborhoods for cognitive function in lower- and middle-income countries where this association may be augmented by limited resources and higher economic inequality. The present study used data from the 2016 Brazilian Longitudinal Study of Aging (ELSI-Brazil) to examine the association between self-reported neighborhood characteristics and cognitive functioning. ELSI-Brazil is a nationally representative sample of community-residing adults in Brazil aged 50 years and older (n = 9,412). We fit a series of linear regression models to test the hypothesis that more favorable neighborhood characteristics are associated with better cognitive functioning. Participants completed self-reported questionnaires of their neighborhood’s social cohesion and availability of healthy foods. A global cognitive score was calculated with a latent variable (g) that was created with confirmatory factor analysis. Adjusted models showed that social cohesion, specifically appraising their neighborhood as a good place to live and trusting most people in their neighborhood, was associated with higher cognitive functioning. Likewise, those who reported greater availability of fresh fruits and vegetables within their neighborhood displayed better cognitive function. Findings provide support for the protective role of neighborhood social cohesion and availability of healthy foods on cognitive function among adults in Brazil. Further research is needed to elucidate what mechanisms may explain this association in the Brazilian context.

